# Genetic Diversity, Population Structure and Mating Type Distribution of *Setosphaeria turcica* on Corn in Midwestern China

**DOI:** 10.3390/jof8111165

**Published:** 2022-11-04

**Authors:** Linkai Cui, Linxi Zhao, Bin Wang, Zanping Han, Yanhong Hu

**Affiliations:** 1 College of Horticulture and Plant Protection, Henan University of Science and Technology, Luoyang 471000, China; 2 College of Agriculture, Henan University of Science and Technology, Luoyang 471000, China

**Keywords:** northern corn leaf blight, *Setosphaeria turcica*, genetic diversity, population structure, mating type, SNP

## Abstract

*Setosphaeria turcica* is the causal agent of northern corn leaf blight (NCLB), which is a destructive foliar disease of corn around the world. To date, limited information is available on the genetic diversity, population structure, and mating type distribution of the pathogen in the mid-west of China. In this study, based on single nucleotide polymorphism (SNP) markers and mating type-specific primers, we characterized 117 *S. turcica* isolates collected from Henan, Hebei, Shanxi, and Shaanxi provinces in China. Based on the developed 33 SNP markers, all isolates can be categorized into two genetic groups. Each group consisted of isolates from all four provinces. The Nei’s gene diversity of four populations ranged from 0.328 to 0.419 with a mean of 0.391. The analysis of fixation index (Fst) and gene flow (Nm) suggested that low genetic differentiation and high gene flow existed among four geographic populations. The analysis of molecular variance (AMOVA) demonstrated that the principal molecular variance existed within populations (98%) rather than among populations (2%). The analysis of mating type loci revealed that two mating types (MAT1-1 and MAT1-2) were basically in equilibrium in all four populations. These findings advance our understanding of the genetic diversity, population structure and mating type distribution of *S. turcica* on corn in the mid-west of China and will aid in developing efficient strategies to control NCLB.

## 1. Introduction

Corn (*Zea mays* L.) is one of the most important crops in the world. More than half of its global production is used for livestock feeds and various industrial raw materials [1]. Northern corn leaf blight (NCLB) caused by *Setosphaeria turcica* (Luttr.) K. J. Leonard & Suggs [anamorph, *Exserohilum turcicum* (Pass.) K. J. Leonard & Suggs] is a devastating foliar disease worldwide [2], which leads to great losses in corn production. In some circumstances, such as low temperature and humid weather, this pathogen can even cause more than 50% yield reduction [3]. In China, NCLB was first reported in 1899 [4] and the disease typically occurs in most corn-growing areas. *S. turcica* causes diseases mainly by inducing necrotic lesions that reduce leaf area available for photosynthesis. During infection of corn by *S. turcica*, leaves first show water-soaked gray-green lesions, and then develop into larger long fusiform necrotic lesions [5]. When the disease is severe, the lesions spots connect with each other, leading to death of the whole plant.

*S. turcica* is a heterothallic ascomycetous fungus, which possesses two mating types, namely MAT1-1 and MAT1-2. Sexual reproduction occurs when the isolates with opposite mating types are in close proximity [6]. The sexual stage of *S. turcica* can be induced under laboratory conditions, and it has also been observed on heavily infected corn leaves from natural fields [7]. Though observations of sexual reproduction in nature are rare, it may be one important source driving genetic variation of the fungal pathogen.

Understanding the genetic diversity and population structure of *S. turcica* is crucial for studying the epidemiology and disease management of NCLB. Various molecular markers, such as random amplified polymorphic DNA (RAPD), amplified fragment length polymorphism (AFLP), sequence-related amplified polymorphism (SRAP), inter-simple sequence repeat (ISSR) and simple sequence repeat (SSR) have been utilized to evaluate the genetic diversity of the pathogen [8,9,10,11,12]. Single nucleotide polymorphism (SNP) is a third-generation molecular marker and has been widely used for studying genetic diversity and population structures of plants and animals, owing to the advantages of ubiquitous presence, uniform distribution, high heritability, and bi-allelic nature [13,14]. However, the SNP marker is rarely applied to plant pathogens at present.

NCLB has occurred seriously and become a major maize disease in the mountainous region of China. Genetic diversity of *S. turcica* on corn was studied in the north and south of China [2,8,12], but so far studies of genetic diversity have been not performed in the middle of China. In this study, we developed SNP markers for *S. turcica* on corn. Based on the SNP markers, the genetic diversity and population structure of *S. turcica* from corn were evaluated in the mid-west of China. In addition, we identified the mating types of *S. turcica* isolates using specific PCR primers and analyzed the ratio of mating types.

## 2. Materials and Methods

### 2.1. Collection and Isolation of S. turcica

The *S. turcica* isolates used in this study were collected from Henan, Hebei, Shanxi and Shaanxi provinces, China, in September 2019 (Supplementary Figure S1). Of 117 isolates, 30 were from Henan, Hebei, and Shanxi, respectively, and 27 were from Shaanxi (Supplementary Table S1). Fungal samples were isolated by the tissue separation method. Briefly, leaves with typical symptoms (Supplementary Figure S2A) were cut into small pieces, treated with 70% ethanol for 1 min, and soaked in 3% sodium hypochlorite solution for another 2 min. After being rinsed three times with sterile water, the leaf fragments were placed on potato dextrose agar (PDA, HuanKai Microbial, Guangzhou, China), amended with 50 μg/mL rifampin (Solarbio, Beijing, China), and incubated at 25 °C for 3 to 5 days. After being examined by light microscopy (Phenix, Shangrao, China), a single hyphal tip of each isolate was picked for another subculture. Purified isolates (Supplementary Figure S2B–E) were stored at 4 °C on PDA slants.

### 2.2. DNA Extraction

*S. turcica* isolates were grown in potato dextrose broth at 25 °C for 3 days. Mycelia of all isolates were collected and dried by sterilized filter papers. DNA extraction was conducted by an Ezup Column Fungi Genomic DNA Purification Kit (Sangon, Shanghai, China) according to the manufacturer’s instructions. The quality and concentration of the gDNA were determined using a NanoDrop 2000 (Thermo Fisher Scientific, Wilmington, DE, USA).

### 2.3. SNP Marker Development

A total of 83,717 SNP loci were identified from whole genome resequencing data of 15 *S. turcica* isolates from corn (https://www.ncbi.nlm.nih.gov/sra/PRJNA834655) (accessed on 3 May 2022) using GATK v4.2.0.0 (Aaron McKenna, Cambridge, MA, USA) [15]. We selected 80 candidate SNP loci that showed a minimal distance of 250 kb from each other. The primers were designed by Batchprimer 3 (https://wheat.pw.usda.gov/demos/BatchPrimer3) (accessed on 29 May 2008) with amplicon sizes ranging from 140 to 270 bp. Based on the 80 SNP loci, 76 SNP primer pairs were successfully designed (Supplementary Table S2) and were commercially synthesized by Sangon Biotech Co., Ltd. (Shanghai, China).

### 2.4. Multiplex PCR and Sequencing

Two panels that contain 76 target SNP sites were designed. One contains 49 target SNP sites, and the other contains 27 target SNP sites. The DNA sequencing library preparation was performed by two-step PCR. Firstly, multiplex PCR was performed based on the two panels. The PCR products were tested by electrophoresis using a 1% (*w/v*) agarose gel (Sangon, Shanghai, China) in TBE buffer, stained with ethidium bromide (EB) and visualized under UV light to determine the size of PCR products, then PCR products were separated from the gel and recovered using AMPure XP magnetic beads (Beckman Coulter, Brea, CA, USA). The second PCR was performed using the PCR products derived from the first round, universal P5 primer and universal P7 primer to obtain the sequencing library with molecular tags. The amplicons were purified by AMPure XP magnetic beads. The specific steps were described by Cui et al. [16]. Finally, paired-end sequencing of the library was performed on the HiSeq XTen sequencers (Illumina, San Diego, CA, USA).

Raw reads were filtered by removing sequencing adapters and low-quality reads using Cutadapt v1.2.1 (NBIS, Dortmund, Germany) [17] and Prinseq-lite v0.20.3 (SOURCEFORGE, Santiago, MN, USA) [18]. The cleaned reads were mapped to the *S. turcica* reference genome (GenBank assembly accession: GCA_013390295.1) using BWA v0.7.13-r1126 (Wheeler Aligner, Cambridge, MA, USA) [19] with default parameters. Samtools v0.1.18 (GitHub, Cambridge, MA, USA) [20] was used to calculate the genotype likelihoods of target sites. The alternate allele frequency was greater than 90%, and SNP loci with missing data were excluded.

### 2.5. Genetic Diversity and Phylogenetic Analysis

In order to analyse the genetic diversity of *S. turcica*, the number of alleles (Na), effective number of alleles (Ne), allele frequencies, Nei’s gene diversity (h) [21] and Shannon’s polymorphism information index (I) [22] were calculated using the program POPGENE v1.31 (Microsoft, Edmonton, AB, Canada) [23]. Phylogenetic analysis was performed by Mega v7.0 (PubMed, Philadelphia, PA, USA) [24] with 1000 bootstrap replications using the neighbor-joining (NJ) method. 

### 2.6. Genetic Differentiation Analysis

In order to analyse the genetic differentiation between populations, the fixation index (Fst) and gene flow (Nm) were calculated with GenAIEx v6.5.2 (Microsoft, Canberra, Australia) [25]. Analysis of molecular variance (AMOVA) was used to apportion the variation within and between populations using the same software.

### 2.7. Population Structure Analysis

Structure V2.3.4 (Palo Alto, Santa Clara, CA, USA) [26] was used to analyze the population structure based on an admixture model. Structure was implemented for K = 1 to 10 with 10 replications after an initial burn-in of 100,000 generations followed by a run length of 1,000,000 generations. The most optimal value of K was identified according to Evanno’s method [27], using Structure Harvester [28].

### 2.8. Mating Type Determination

Mating types of *S. turcica* isolates were examined by multiple PCR assays on the basis of the mating type-specific primers reported by Haasbroek M.P. [29]. The *S. turcica* MAT1-1 locus and MAT1-2 locus were amplified using the same reverse primer (MAT_CommonR, 5′-AATGCGGACACGGAATAC-3′). Forward primer MAT_1-1F (5′-CTCGTCCTTGGAGAAGAATATC-3′) was employed for MAT1-1, and MAT_1-2F (5′-GCTCCTGGACCAAATAATACA-3′) for MAT1-2. PCR was performed as described previously [16]. The mating type-specific primer pairs generated 608 bp amplicons of MAT1-1 and 393 bp of MAT1-2. Ratios between MAT1-1 and MAT1-2 from each geographical population were subjected to a χ^2^ test using IBM SPSS Statistics 20 (International Business Machines Corporation, Arkmonk, NY, USA) to evaluate divergence from the expected ratio of 1:1 at the *p* < 0.05 level.

## 3. Results

### 3.1. Genetic Diversity

Based on 80 candidate SNP loci, 76 primer pairs were successfully designed by Batchprimer 3. A total of 33 primer pairs amplified the expected DNA bands from all 117 isolates, and the 33 SNP loci were polymorphic (Supplementary Table S3). The observed number of alleles at each locus were two, so all SNP markers were biallelic markers (Table 1). The number of effective alleles per locus ranged from 1.246 to 1.999, with a mean of 1.733. The minor allele frequency (MAF) ranged from 0.111 to 0.495, with 70% of the markers having MAF > 0.2. The Nei’s gene diversity showed a range of 0.198 to 0.5, with a mean of 0.406. Shannon’s information index varied from 0.349 to 0.693, with a mean of 0.593. Thirty-three SNP markers were successfully developed for genetic diversity analysis of *S. turcica*.

At the population level, the Nei’s gene diversities were 0.419 (Henan), 0.412 (Shaanxi), 0.404 (Hebei) and 0.328 (Shanxi), with an average of 0.391 (Table 2). Shannon’s information indices were 0.625, 0.599, 0.587 and 0.504 for Henan, Shaanxi, Hebei, and Shanxi populations, respectively. The genetic diversity of the Shanxi population was lower than the other three populations, and the genetic diversity of the Henan population was the most abundant.

### 3.2. Phylogenetic Analysis

**Figure 1:**
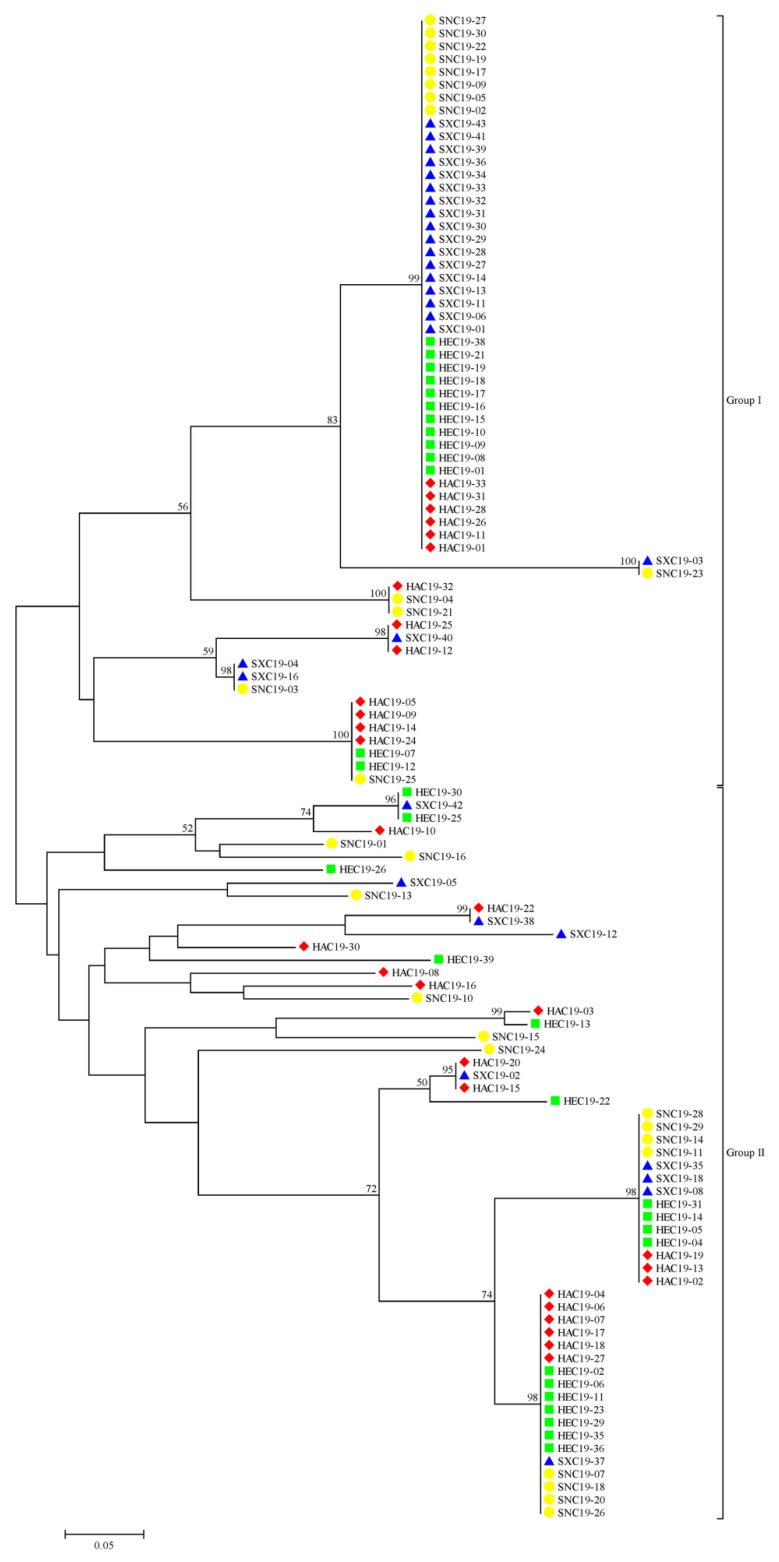
Phylogenetic tree of 117 *Setosphaeria turcica* isolates based on the neighbor-joining method. The red rhombus, green square, blue triangle, and yellow circle represent the Henan, Hebei, Shanxi, and Shaanxi populations, respectively.

Using the neighbor-joining method, 117 *S. turcica* isolates were categorized into two groups (Figure 1). There were 60 isolates in group I, including 13 isolates from Henan, 13 isolates from Hebei, 21 isolates from Shanxi, and 13 isolates from Shaanxi. There were 57 isolates in group II, including 17 isolates from Henan, 17 isolates from Hebei, 9 isolates from Shanxi, and 14 isolates from Shaanxi. These isolates from four geographic populations were divided into one group, and even into one clade. These results suggested that the migration of *S. turcica* could happen frequently among the four provinces in China and there was no significant correlation between genetic groups and geographic origin.

### 3.3. Genetic Differentiation

Genetic differentiation among populations was estimated using Fst. The Fst values among the four populations of *S. turcica* ranged from 0.006 to 0.057 (Table 3), suggesting that there existed some genetic differentiation among the four populations, but the genetic differentiation was relatively low. Gene flow analysis showed that the Nm values among the four populations of *S. turcica* varied from 6.647 to 45.148 (Table 3). All values of Nm exceeded 6, suggesting that high gene flow existed between the pairwise populations of the four geographic populations. The AMOVA analysis showed that 98% of the total genetic variation was distributed within the four populations (Table 4). The remaining 2% of the genetic variance was caused by geographic separation among the four geographic populations.

### 3.4. Population Structure

**Figure 2:**
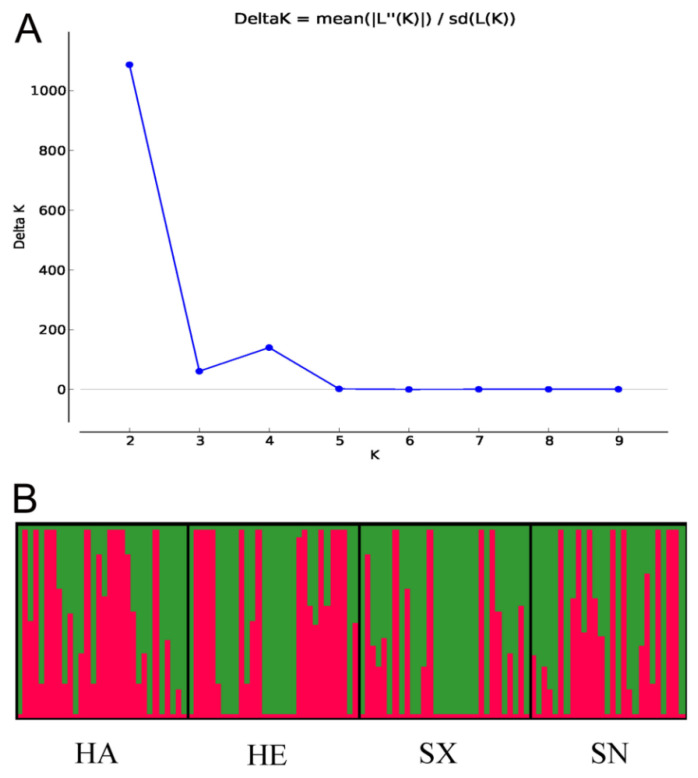
Graphical representation of the results obtained from STRUCTURE. (**A**) DeltaK curve showing evidence of two genetic clusters. (**B**) STRUCTURE plot of 117 *Setosphaeria turcica* isolates with K = 2. Each vertical line represents an isolate. Boxes framed by thick black lines indicate different geographic populations.

The Delta K value was highest at K = 2, suggesting that two genetic clusters best explained the population structure of *S. turcica* isolates from four geographic populations in China (Figure 2). Though each population consisted of two genetic clusters in the four geographic populations, the main source of clusters in every population was slightly different. The proportion of the red cluster was more in the Henan population, while the proportion of the green cluster was more in the Shanxi population. The two clusters were relatively evenly distributed in Hebei and Shaanxi populations. These results indicated that there was a low degree of genetic differentiation among the four populations.

### 3.5. Mating Type Distribution

Based on the mating type-specific primers, the mating type idiomorph was successfully amplified in all 117 *S. turcica* isolates and all isolates had only either the MAT1-1 or MAT1-2 mating type idiomorph. Forty-five isolates were determined as MAT1-1, and the remaining 72 isolates were determined as MAT1-2. The ratio of MAT1-1 and MAT1-2 for all isolates did not differ significantly from a 1:1 ratio (*p* = 0.8, Table 5). For each geographic population, the frequencies of mating type also did not deviate significantly from a 1:1 ratio (0.1 ≤ *p* ≤ 0.6, Table 5). These results suggested that sexual reproduction of *S. turcica* could be occurring in the four populations of China.

## 4. Discussion

In this study, 33 SNP markers were developed for *S. turcica* on corn based on 80 SNP loci. In our previous study, 36 SNP markers were developed for *S. turcica* on sorghum based on 80 SNP loci [16]. Of these SNP loci, 50 SNP loci were identical for the two studies. In the 50 SNP loci, 13 SNP markers were developed for *S. turcica* on corn and 12 SNP markers were developed for *S. turcica* on sorghum. Only 7 SNP markers can be used not only for *S. turcica* on corn but also for *S. turcica* on sorghum. Six SNP markers can be used only for *S. turcica* on corn and 5 SNP markers can be used only for *S. turcica* on sorghum, suggesting that genetic difference between *S. turcica* on corn and *S. turcica* on sorghum is significant.

Genetic diversity is the basis of biological diversity research, which reflects the adaptability of populations and the potential to be transformed and utilized [30]. Nei’s gene diversity (h) is an important parameter for measuring genetic diversity of a population [31]. In the present study, Nei’s gene diversity (h) ranged from 0.328 to 0.419 with a mean of 0.391, indicating that the genetic diversity of *S. turcica* was relatively low in the mid-west of China. In the recent studies, Ma et al. [11] reported that low genetic diversity (h = 0.2634) was detected within the *S. turcica* populations in the north of China, and Dai et al. [12] reported that the Fujian population of *S. turcica* showed a low gene diversity (h = 0.13) in the south of China. These findings suggest that there is a low gene diversity in the Chinese populations of *S. turcica*. The result is consistent with previous findings of genetic diversity of *S. turcica* in other countries. Human et al. [10] reported low genetic diversity of *S. turcica* in South Africa, and Turgay et al. [32] also reported low genetic diversity of *S. turcica* in Turkey.

Gene flow is an important factor that can reduce geographic differentiation and result in the homogenization of genetic characteristics among populations [33]. In our study, a high degree of gene flow was detected among the four geographic populations of *S. turcica*. Henan, Shaanxi, Hebei, and Shanxi are four neighboring provinces in the mid-west of China. The migration of *S. turcica* among the four provinces should be main cause of high gene flow, which is supported by the phylogenetic analysis that *S. turcica* isolates from four geographic populations were divided into one clade. Gene flow homogenizes populations by genetically decreasing variance among populations and increasing variance within populations. So a low level of genetic differentiation was detected among the four geographic populations of *S. turcica*, which is consistent with the AMOVA analysis that the principal molecular variance existed within populations rather than among populations.

Understanding the distribution of the mating types of a phytopathogen is essential to infer the level of population genetic variability attributable to the underlying sexual recombination [34]. In our study, MAT1-2 was the predominant mating type in the four geographic populations, but the ratio of MAT1-1 and MAT1-2 did not differ significantly from a 1:1 ratio (*p* < 0.05), implying that sexual reproduction of *S. turcica* probably occurs under natural conditions in the four provinces of China. Sexual reproduction has been hypothesized as a critical factor causing high genetic variation in plant fungal pathogens [35,36]. However, a relatively low genetic diversity was detected in the four geographic populations. This may be because sexual reproduction of *S. turcica* occurs at a low frequency and asexual reproduction is still the main reproductive mode. At present, sexual reproduction of *S. turcica* has not been observed in natural corn fields of China. In future, we will investigate the sexual reproduction of *S. turcica* in order to figure out its role in Chinese populations.

## Figures and Tables

**Table 1:** Characteristics of 33 SNP markers.

Locus	Ref	Alt	Allele I	Allele II	Na	Ne	h	I
L01	C	T	0.5043	0.4957	2	1.9999	0.5000	0.6931
L02	G	T	0.5812	0.4188	2	1.9486	0.4868	0.6799
L03	A	G	0.8547	0.1453	2	1.3304	0.2484	0.4145
L07	G	A	0.4872	0.5128	2	1.9987	0.4997	0.6928
L12	G	A	0.1624	0.8376	2	1.3737	0.2720	0.4436
L14	T	C	0.4786	0.5214	2	1.9964	0.4991	0.6922
L16	C	G	0.8462	0.1538	2	1.3520	0.2604	0.4293
L17	C	T	0.5726	0.4274	2	1.9586	0.4894	0.6826
L19	G	A	0.4274	0.5726	2	1.9586	0.4894	0.6826
L20	T	C	0.3162	0.6838	2	1.7620	0.4325	0.6240
L21	A	C	0.1453	0.8547	2	1.3304	0.2484	0.4145
L25	C	T	0.8120	0.1880	2	1.4396	0.3054	0.4834
L26	G	T	0.1111	0.8889	2	1.2462	0.1975	0.3488
L27	G	A	0.4701	0.5299	2	1.9929	0.4982	0.6914
L29	C	A	0.6581	0.3419	2	1.8182	0.4500	0.6423
L30	G	A	0.6239	0.3761	2	1.8842	0.4693	0.6621
L31	C	T	0.6410	0.3590	2	1.8526	0.4602	0.6528
L33	G	A	0.1795	0.8205	2	1.4175	0.2945	0.4706
L34	G	T	0.4786	0.5214	2	1.9964	0.4991	0.6922
L36	C	T	0.3675	0.6325	2	1.8688	0.4649	0.6576
L37	G	A	0.8376	0.1624	2	1.3737	0.2720	0.4436
L39	A	G	0.2308	0.7692	2	1.5505	0.3550	0.5402
L40	G	A	0.1795	0.8205	2	1.4175	0.2945	0.4706
L41	C	T	0.4103	0.5897	2	1.9376	0.4839	0.6770
L45	G	A	0.4786	0.5214	2	1.9964	0.4991	0.6922
L47	C	T	0.6923	0.3077	2	1.7423	0.4260	0.6172
L48	G	A	0.8376	0.1624	2	1.3737	0.2720	0.4436
L51	T	C	0.5641	0.4359	2	1.9677	0.4918	0.6849
L58	C	T	0.4188	0.5812	2	1.9486	0.4868	0.6799
L60	G	A	0.2308	0.7692	2	1.5505	0.3550	0.5402
L61	G	A	0.4701	0.5299	2	1.9929	0.4982	0.6914
L62	C	G	0.4615	0.5385	2	1.9882	0.4970	0.6902
L65	T	C	0.3419	0.6581	2	1.8182	0.4500	0.6423
Mean					2	1.7328	0.4075	0.5928

Note: Na, Number of observed alleles; Ne, Number of effective alleles; h, Nei’s gene diversity reported; I, Shannon’s information index.

**Table 2:** Genetic diversity of *Setosphaeria turcica* populations from Henan, Hebei, Shanxi and Shaanxi in China.

Population	Na	Ne	h	I
Henan	2	1.764	0.419	0.625
Hebei	2	1.733	0.404	0.587
Shanxi	2	1.519	0.328	0.504
Shaanxi	2	1.741	0.412	0.599
Mean	2	1.689	0.391	0.574

Note: Na, Number of observed alleles; Ne, Number of effective alleles; h, Nei’s gene diversity reported; I, Shannon’s information index.

**Table 3:** Pairwise matrices of Fst (below the diagonal) and Nm (above the diagonal) among four populations of *Setosphaeria turcica* in China.

Population	Henan	Hebei	Shanxi	Shaanxi
Henan		22.025	4.131	19.116
Hebei	0.011		6.647	45.148
Shanxi	0.057	0.036		8.054
Shaanxi	0.013	0.006	0.030	

**Table 4:** Molecular variance analysis within and among four populations of *Setosphaeria turcica* in China.

Source	Degree of Freedom	Sum of Squares	Variation (%)	*p*
Among Pops	3	133.754	2%	0.091
Within Pops	113	3021.015	98%	0.091
Total	116	3154.769	100%	

**Table 5:** Mating type distribution in four Chinese populations of *Setosphaeria turcica*.

Population	MAT1-1	MAT1-2	χ^2^	*p*
Henan	13	17	0.27	0.6
Hebei	12	18	0.61	0.4
Shanxi	9	21	2.5	0.1
Shaanxi	11	16	0.47	0.5
Total	45	72	3.16	0.8

## Data Availability

Data may be accessed by contacting the corresponding author.

## Supplementary Materials

The following supporting information can be downloaded at: https://www.mdpi.com/article/10.3390/jof8111165/s1, Figure S1: Map illustrating the location of four provinces in which *Setosphaeria turcica* isolates were collected. The blue regions denote coastlines or islands. HA = Henan province, HE = Hebei province, SX = Shanxi province, SN = Shaanxi province; Figure S2: Northern corn leaf blight and *Setosphaeria turcica*. (A) Typical symptoms of northern corn leaf blight. (B) The fungal colony (upper surface). (C) The fungal colony (lower surface). (D) Conidium. (E) Conidiophore; Table S1: *Setosphaeria turcica* isolates used in this study; Table S2: 76 SNP loci and primer pairs for *Setosphaeria turcica*. Table S3: 33 SNP loci and primer pairs used to analyze genetic diversity of *Setosphaeria turcica* populations in China.
