# Supplementary material for: Genetic Diversity, Population Structure and Mating Type Distribution of Setosphaeria turcica on Corn in Midwestern China
Source: J Fungi (Basel). 2022 Nov 4;8(11):1165. doi: 10.3390/jof8111165 (PMC9694368; doi:10.3390/jof8111165)
Supplement: Supplementary file 1 [file jof-08-01165-s001.zip › jof-1946790-supplementary.pdf]

Journal of Fungi

MDPI

Table S1. *Setosphaeria turcica* isolates used in this study.

Isolate	Location	Year	Mating Type	Isolate	Location	Year	Mating Type
HAC19-01	Jiyuan, Henan	2019	MAT1-2	HEC19-39	Handan, Hebei	2019	MAT1-2
HAC19-02	Jiyuan, Henan	2019	MAT1-1	SXC19-01	Yuncheng, Shanxi	2019	MAT1-2
HAC19-03	Jiyuan, Henan	2019	MAT1-2	SXC19-02	Yuncheng, Shanxi	2019	MAT1-2
HAC19-04	Jiyuan, Henan	2019	MAT1-1	SXC19-03	Yuncheng, Shanxi	2019	MAT1-2
HAC19-05	Jiyuan, Henan	2019	MAT1-2	SXC19-04	Yuncheng, Shanxi	2019	MAT1-1
HAC19-06	Jiyuan, Henan	2019	MAT1-1	SXC19-05	Yuncheng, Shanxi	2019	MAT1-2
HAC19-07	Jiyuan, Henan	2019	MAT1-1	SXC19-06	Yuncheng, Shanxi	2019	MAT1-2
HAC19-08	Jiyuan, Henan	2019	MAT1-2	SXC19-08	Yuncheng, Shanxi	2019	MAT1-1
HAC19-09	Sanmenxia, Henan	2019	MAT1-2	SXC19-11	Yuncheng, Shanxi	2019	MAT1-2
HAC19-10	Sanmenxia, Henan	2019	MAT1-1	SXC19-12	Yuncheng, Shanxi	2019	MAT1-1
HAC19-11	Sanmenxia, Henan	2019	MAT1-2	SXC19-13	Yuncheng, Shanxi	2019	MAT1-2
HAC19-12	Sanmenxia, Henan	2019	MAT1-1	SXC19-14	Yuncheng, Shanxi	2019	MAT1-2
HAC19-13	Sanmenxia, Henan	2019	MAT1-1	SXC19-16	Yuncheng, Shanxi	2019	MAT1-1
HAC19-14	Sanmenxia, Henan	2019	MAT1-2	SXC19-18	Yuncheng, Shanxi	2019	MAT1-1
HAC19-15	Sanmenxia, Henan	2019	MAT1-2	SXC19-27	Jinzhong, Shanxi	2019	MAT1-2
HAC19-16	Sanmenxia, Henan	2019	MAT1-2	SXC19-28	Jinzhong, Shanxi	2019	MAT1-2
HAC19-17	Sanmenxia, Henan	2019	MAT1-1	SXC19-29	Jinzhong, Shanxi	2019	MAT1-2
HAC19-18	Sanmenxia, Henan	2019	MAT1-1	SXC19-30	Jinzhong, Shanxi	2019	MAT1-2
HAC19-19	Sanmenxia, Henan	2019	MAT1-1	SXC19-31	Jinzhong, Shanxi	2019	MAT1-2
HAC19-20	Sanmenxia, Henan	2019	MAT1-2	SXC19-32	Jinzhong, Shanxi	2019	MAT1-2
HAC19-22	Sanmenxia, Henan	2019	MAT1-1	SXC19-33	Jinzhong, Shanxi	2019	MAT1-2
HAC19-24	Sanmenxia, Henan	2019	MAT1-2	SXC19-34	Jinzhong, Shanxi	2019	MAT1-2
HAC19-25	Sanmenxia, Henan	2019	MAT1-1	SXC19-35	Linfen, Shanxi	2019	MAT1-1
HAC19-26	Sanmenxia, Henan	2019	MAT1-2	SXC19-36	Linfen, Shanxi	2019	MAT1-2
HAC19-27	Sanmenxia, Henan	2019	MAT1-1	SXC19-37	Linfen, Shanxi	2019	MAT1-1
HAC19-28	Luoyang, Henan	2019	MAT1-2	SXC19-38	Linfen, Shanxi	2019	MAT1-1
HAC19-30	Luoyang, Henan	2019	MAT1-2	SXC19-39	Linfen, Shanxi	2019	MAT1-2
HAC19-31	Luoyang, Henan	2019	MAT1-2	SXC19-40	Linfen, Shanxi	2019	MAT1-1
HAC19-32	Luoyang, Henan	2019	MAT1-2	SXC19-41	Linfen, Shanxi	2019	MAT1-2
HAC19-33	Luoyang, Henan	2019	MAT1-2	SXC19-42	Linfen, Shanxi	2019	MAT1-2
HEC19-01	Handan, Hebei	2019	MAT1-2	SXC19-43	Linfen, Shanxi	2019	MAT1-2
HEC19-02	Handan, Hebei	2019	MAT1-1	SNC19-01	Xi'an, Shaanxi	2019	MAT1-2
HEC19-04	Handan, Hebei	2019	MAT1-1	SNC19-02	Xianyang, Shaanxi	2019	MAT1-2
HEC19-05	Handan, Hebei	2019	MAT1-1	SNC19-03	Xianyang, Shaanxi	2019	MAT1-1
HEC19-06	Handan, Hebei	2019	MAT1-1	SNC19-04	Xianyang, Shaanxi	2019	MAT1-2
HEC19-07	Handan, Hebei	2019	MAT1-2	SNC19-05	Xianyang, Shaanxi	2019	MAT1-2
HEC19-08	Handan, Hebei	2019	MAT1-2	SNC19-07	Xianyang, Shaanxi	2019	MAT1-1
HEC19-09	Handan, Hebei	2019	MAT1-2	SNC19-09	Xianyang, Shaanxi	2019	MAT1-2
HEC19-10	Handan, Hebei	2019	MAT1-2	SNC19-10	Xianyang, Shaanxi	2019	MAT1-2
HEC19-11	Handan, Hebei	2019	MAT1-1	SNC19-11	Xianyang, Shaanxi	2019	MAT1-1
HEC19-12	Handan, Hebei	2019	MAT1-2	SNC19-13	Weinan, Shaanxi	2019	MAT1-2
HEC19-13	Handan, Hebei	2019	MAT1-2	SNC19-14	Weinan, Shaanxi	2019	MAT1-1
HEC19-14	Handan, Hebei	2019	MAT1-1	SNC19-15	Weinan, Shaanxi	2019	MAT1-1
HEC19-15	Handan, Hebei	2019	MAT1-2	SNC19-16	Weinan, Shaanxi	2019	MAT1-2
HEC19-16	Xingtai, Hebei	2019	MAT1-2	SNC19-17	Weinan, Shaanxi	2019	MAT1-2
HEC19-17	Xingtai, Hebei	2019	MAT1-2	SNC19-18	Weinan, Shaanxi	2019	MAT1-1
HEC19-18	Xingtai, Hebei	2019	MAT1-2	SNC19-19	Weinan, Shaanxi	2019	MAT1-2
HEC19-19	Xingtai, Hebei	2019	MAT1-2	SNC19-20	Weinan, Shaanxi	2019	MAT1-1

HEC19-21	Handan, Hebei	2019	MAT1-2	SNC19-21	Weinan, Shaanxi	2019	MAT1-2
HEC19-22	Handan, Hebei	2019	MAT1-2	SNC19-22	Weinan, Shaanxi	2019	MAT1-2
HEC19-23	Handan, Hebei	2019	MAT1-1	SNC19-23	Weinan, Shaanxi	2019	MAT1-2
HEC19-25	Handan, Hebei	2019	MAT1-2	SNC19-24	Huayin, Shaanxi	2019	MAT1-1
HEC19-26	Handan, Hebei	2019	MAT1-1	SNC19-25	Huayin, Shaanxi	2019	MAT1-2
HEC19-29	Handan, Hebei	2019	MAT1-1	SNC19-26	Huayin, Shaanxi	2019	MAT1-1
HEC19-30	Handan, Hebei	2019	MAT1-2	SNC19-27	Huayin, Shaanxi	2019	MAT1-2
HEC19-31	Handan, Hebei	2019	MAT1-1	SNC19-28	Huayin, Shaanxi	2019	MAT1-1
HEC19-35	Handan, Hebei	2019	MAT1-1	SNC19-29	Huayin, Shaanxi	2019	MAT1-1
HEC19-36	Handan, Hebei	2019	MAT1-1	SNC19-30	Huayin, Shaanxi	2019	MAT1-2
HEC19-38	Handan, Hebei	2019	MAT1-2				

Table S2. 76 SNP loci and primer pairs for *Setosphaeria turcica.*

Locus	Contig-SNP Position	Forward Primer (5'-3')	Reverse Primer (5'-3')
L01	1-532966	CACCGCACAGTCCTCAACTTT	TTAAAATTTGGCTTACCAGGACTTG
L02	1-2258635	GCTACCTCTCGAAGAAAAGGAAGTC	TACCAGCTTTCTCAAAGACATCAAG
L03	1-2799230	TAGTTTAACGTCCTTTTTCAGCCC	GTAACAGGTAGCCATACACCTCCC
L04	2-51559	AAGAGCTTGCCTTGTTTGAGAGTTA	CTAAGCTCTAGCACGAGTACGAAGG
L05	2-1233702	TAGCTGCGATCCACGAGTTAAGTA	GATGACTAGGCCATGTCGAAAAGT
L06	2-2069656	TCAAGTTTGGAACGTTAATCTCCAT	TAGAACCGGCTAGAAGAGAAGTACG
L07	2-3138855	CTAAGTTTAAAGAGGACGTGATTGT	TCAACGTTTAACAAAGGTACTAGGC
L08	3-86782	GCAGAAATCTTTCATCAAAACTCGT	AAGAATACCGACAATACCCCCTCTA
L09	3-976275	CCTGGTCGATATAGCAGCTCTAGG	GGAATGGGATTCTAAGCTACGTTT
L10	3-1859865	ACGACGTACTAATCTACACCGATGG	CATGCTTATCCCTTTACTTGCTTTG
L11	3-2767063	GGTGTAATTTACTGCCCAGGATCT	GATAGCCTTTGTCGGCAGAATAGT
L12	4-395896	AGAGAAAGAAGAAAGAAGAAAGAAGAGC	CTGTAGTTGTTAAACGTGGAAAAGACAT
L13	4-1195115	AGATAAAAATGCTCTTCTTAGCGGG	TTAAATAATTAAGGGGGTGAATGGC
L14	4-2013962	TCAGTATCCCAACATCAAGTACGAA	CTGAAAGTCTTGTCTGACATGTTGC
L15	4-2818621	AGAGATAGAGTCACAAGGAACGCC	GACATCATTTCCTAGTGTGCGACT
L16	5-118908	GTTAGGCTTAAGAGAGAAGGCACA	GACCTCCTAAACTTAAGAGCTTAGCC
L17	5-788041	ATATACCCCGCAGTATCACTTCAAA	CCACGTATGACCTCTTCTTTGCTA
L18	5-1616899	GCACCAAAGACAGTAAAGGGAGTAA	TGTAAGTCTAGTAAGAGGTGCGGCT
L19	5-2306034	TGAATCTCGCAAACTATTGCCTAC	TCTCCTAAGGCACTACAAGTTCTACC
L20	6-356581	TGAATGTCAACGTCCAAACTCAAC	CTATAAGGGTGTGTGGCGGGTTAT
L21	6-1089782	ACACTGTTTCAGATAAAGCTGGTGC	TAATGAAGCTGCCTTCTACTTACCG
L22	6-1690306	GCTCTTAGGTTTTCTTACGTAACTCTT	GCCATAGACAACGCTTAAAAGAT
L23	6-2202372	AAGGAGCTCAGCAGCAACAAG	GCACCTCCTTGCTTAGCACC
L24	7-418160	CCTTCGTAGGCTTCTCTTAGTCCT	CTAGGGCACTGTTACCTAAGGGAC
L25	7-852881	CCCACACTCCGACGATTACTACTAC	CAGTGTGTTTTCTGCGTCTCG
L26	7-1504161	TCTACAGCTGCACTAACTAGGGTCC	AAAGACATGTTGATTGTGTGGTGTT
L27	7-2101471	ATCTGCCTAAGGACTGCGTTGTAT	TAGGCTAAAATGCTCGCCTAATTT
L28	8-311165	CAAGTCTATGCCCCACTTATTCCT	AGACGTTGCGTATGCTATTTCCTA
L29	8-824392	CGTTTTAACAAAAAGGTTTACGGC	CTCTGTTGGTACTCTGCTGAAGTTG
L30	8-1414857	GACAAACGGTGAGATGGCCTA	CACATCGACAGCACAACTTCC
L31	9-153530	GAGAGAATCCTTGGTGAAGAGAAAA	GTTGGCTCGATAAGCTCCATATCC
L32	9-549664	AAAGTTTTGTCCCACGCTAATCTC	ACTTTAACGCCACAATTAAGGTGC
L33	9-1137846	CGACCATTGAAGTTATTAGCAGGAT	GCTGTGAAGATCTTTGCTCAAGATAA
L34	9-1664862	GTTAAGAAGTGCTTGGGTAGGTCG	GCAGGCCTAAGAGAAAGAAGAATC

L35	10-504107	TTCTACGTCTTCTGCTAGTTCAGC	GACTATTTAAAATCCTAGAGAAGGTAGG
L36	10-1007850	GTAGAGATTGCTATCAAGACCCTCG	CTACCAAGAATTGTCAGTACAGGGC
L37	10-1504344	CTTACTAGGCGTCTGTTGTTGGACT	GAATTAGTCTTCGATCTAAACGCCA
L38	10-1978944	GCATGCATTTGATGACTAACGACT	ATGCTGTCGACCACACTCAACATA
L39	11-88127	TAGCAAAGTGTTGGAGAAGTGTGAG	TATCCTTGAATTTGGAGTCTTGGTG
L40	11-780809	GCCTCTTAATTTCTCTAAGCGTAATAGC	GCAGCCCCTTACACTACGTAAGAA
L41	11-1660295	TGGCTTTTTATGGTGTACAAGACC	CAGACCAAGTCAGTCTCGACGTAT
L42	12-459017	CCCCCTACTTATGATCCAAGCTAA	CCCACATACGCCATGTAAAATAAA
L43	12-1366430	CCTTAGTTTTGATGGTTTAAGAGGC	TAAGCTGTATTCGGTTATTTGCGA
L44	13-198147	CCTCTTATATACCTCGCGATTGCT	CGTACCTATTGCAGCACTAACTCTG
L45	13-887198	TCAAAGTTACACTGGTATATGGCGA	ACTTGGCCTCCGATGTAAGTGT
L46	13-1528663	CTATTGGAGACGGTGATCGACAT	ATAAATCTGCGACACGGCTTTT
L47	14-310822	AAGAATAACGCGATAAGCGAAGTC	GCTTGGCATCTCTACCTATTCGAT
L48	14-759423	GCTCTATCCATTCCATTGACATCTT	ATAAATGGTGGGAAGAGGGTGTAGT
L49	14-1206381	AGCATGACGTTAAGCTGTGATAAAA	AGCAAAGAAACCTTCCTACTAGCG
L50	15-481165	CAAAAGAAATTGTTGCGTCGTAAG	AAAAGACTAATCCAAAGTAGCCGC
L51	15-845551	TATCAACAAGAACCCACATTACCCT	GTATTAGCAGACGACGACATATCCC
L52	15-1342388	TGCAGCATCACTATCTTGGAAACT	GTTTCATGTGCCATAACAACATCG
L53	16-248152	GTTCTCTAAGCGTTTTGCTAGATG	GAGCTATGGTGGAAAAAGAGATCC
L54	16-866400	ATAGTACTGCTATTGTGCTGCGGTT	CCCTGCTGTCTAACATCTCTTCCT
L55	16-1240230	AGGTGAATGCATACTCGTTAGTTAGG	TGCCTTATACAAAAGATCCTTACGC
L56	17-287484	GGAGTAACCGGACTACCTTTTCAA	AGGCTTTCTGTTAGCTCGATCACT
L57	17-831816	AAACGGCTACAAGATGGCG	ATAGCAAGTACAGCTGCAGAATGG
L58	17-1112393	GAAGAATGGAGTTTCTCATTTCACG	CCGGGTCTAAACTCGTATTTCTCTT
L59	18-111419	TTTATTCCTCTCGTCCTCATCCTC	GTATCATTTCGGTTTTCCAGGTTC
L60	18-593631	AATTGAGCAAGTGGTATGCGTG	CATTCCATCATTCATCTACTCACCA
L61	19-71784	GGACAAGGTCACGCTAGTAAAGGT	TAGGTGAGATTATCGTTCGAGCAG
L62	19-461680	CTAGCCACACGCTCTATCCACAG	GTCTGCTGAGGCTCGAAATGAT
L63	19-779640	ATTGAGGCTGTATCCCGCAGT	GCTGGTCCTCAGTCCAAAGTATCT
L64	20-131994	ACAAACAAAACCAGCGTCACTTT	AACACCCCTCGTGTGTGATATTTT
L65	20-447203	TGTCGAGCTTGTTATATTAACGGAA	AACACCATTACCTACAGTCTCGTCG
L66	20-829679	AAAGCTGCGTACGCCTACTACTCT	GCCCCCTTCTTAAGCTAGTTAATTG
L67	21-14063	GCGCGTTATTATCTATATATCAGCTCT	GACTAAAGCTAAGCTACTTTTATTGGAG
L68	21-458974	TGCAGTCCAGTGATGTATGTATGC	GCCTGGAATAATGGGAGATTGTAG
L69	22-56000	CGCTATTTAGAGCGTTCTTATGCC	TACCTTAGCAGGGGATGGATAGAC
L70	22-560832	CGCTGCATGATCTAATTACAAAGG	GGGTTTTAGCTTCTCGATTGATCT
L71	23-87112	GATGATCTTGCGACTTACTGCCTA	TGTTTTAGTTTGGCTCTCCCTCTT
L72	24-484964	TTTTTGTACCAGTAGCAGCCGAC	CATTCAGACCTCGTAACCAAACAC
L73	26-263600	TAAAGCACTTTGTCAAGCATACGG	GCTGGAGTAGGTGAGATGAGTGAG
L74	27-91353	AGTCTTTTACTGTACGGTCCACCC	AAGAGAAGGTGATCAGAGCTTTCG
L75	28-48379	TGGGCGTACTATTGGTTAATCCTT	ATAGCACTTCGCAAACAATCTCAC
L76	29-25741	AAGCGCTTTGGTGTACATGCT	AGCTTACATTCACCGCACTCAAC

Table S3. 33 SNP loci and primer pairs used to analyze genetic diversity of *Setosphaeria turcica* populations in China.

Locus	Contig-SNP Position	Forward Primer (5'-3')	Reverse Primer (5'-3')
L01	1-532966	CACCGCACAGTCCTCAACTTT	TTAAAATTTGGCTTACCAGGACTTG
L02	1-2258635	GCTACCTCTCGAAGAAAAGGAAGTC	TACCAGCTTTCTCAAAGACATCAAG
L03	1-2799230	TAGTTTAACGTCCTTTTTCAGCCC	GTAACAGGTAGCCATACACCTCCC
L07	2-3138855	CTAAGTTTAAAGAGGACGTGATTGT	TCAACGTTTAACAAAGGTACTAGGC
L12	4-395896	AGAGAAAGAAGAAAGAAGAAAGAAGAGC	CTGTAGTTGTTAAACGTGGAAAAGACAT
L14	4-2013962	TCAGTATCCCAACATCAAGTACGAA	CTGAAAGTCTTGTCTGACATGTTGC
L16	5-118908	GTTAGGCTTAAGAGAGAAGGCACA	GACCTCCTAAACTTAAGAGCTTAGCC
L17	5-788041	ATATACCCCGCAGTATCACTTCAAA	CCACGTATGACCTCTTCTTTGCTA
L19	5-2306034	TGAATCTCGCAAACTATTGCCTAC	TCTCCTAAGGCACTACAAGTTCTACC
L20	6-356581	TGAATGTCAACGTCCAAACTCAAC	CTATAAGGGTGTGTGGCGGGTTAT
L21	6-1089782	ACACTGTTTCAGATAAAGCTGGTGC	TAATGAAGCTGCCTTCTACTTACCG
L25	7-852881	CCCACACTCCGACGATTACTACTAC	CAGTGTGTTTTCTGCGTCTCG
L26	7-1504161	TCTACAGCTGCACTAACTAGGGTCC	AAAGACATGTTGATTGTGTGGTGTT
L27	7-2101471	ATCTGCCTAAGGACTGCGTTGTAT	TAGGCTAAAATGCTCGCCTAATTT
L29	8-824392	CGTTTTAACAAAAAGGTTTACGGC	CTCTGTTGGTACTCTGCTGAAGTTG
L30	8-1414857	GACAAACGGTGAGATGGCCTA	CACATCGACAGCACAACTTCC
L31	9-153530	GAGAGAATCCTTGGTGAAGAGAAAA	GTTGGCTCGATAAGCTCCATATCC
L33	9-1137846	CGACCATTGAAGTTATTAGCAGGAT	GCTGTGAAGATCTTTGCTCAAGATAA
L34	9-1664862	GTTAAGAAGTGCTTGGGTAGGTCG	GCAGGCCTAAGAGAAAGAAGAATC
L36	10-1007850	GTAGAGATTGCTATCAAGACCCTCG	CTACCAAGAATTGTCAGTACAGGGC
L37	10-1504344	CTTACTAGGCGTCTGTTGTTGGACT	GAATTAGTCTTCGATCTAAACGCCA
L39	11-88127	TAGCAAAGTGTTGGAGAAGTGTGAG	TATCCTTGAATTTGGAGTCTTGGTG
L40	11-780809	GCCTCTTAATTTCTCTAAGCGTAATAGC	GCAGCCCCTTACACTACGTAAGAA
L41	11-1660295	TGGCTTTTTATGGTGTACAAGACC	CAGACCAAGTCAGTCTCGACGTAT
L45	13-887198	TCAAAGTTACACTGGTATATGGCGA	ACTTGGCCTCCGATGTAAGTGT
L47	14-310822	AAGAATAACGCGATAAGCGAAGTC	GCTTGGCATCTCTACCTATTCGAT
L48	14-759423	GCTCTATCCATTCCATTGACATCTT	ATAAATGGTGGGAAGAGGGTGTAGT
L51	15-845551	TATCAACAAGAACCCACATTACCCT	GTATTAGCAGACGACGACATATCCC
L58	17-1112393	GAAGAATGGAGTTTCTCATTTCACG	CCGGGTCTAAACTCGTATTTCTCTT
L60	18-593631	AATTGAGCAAGTGGTATGCGTG	CATTCCATCATTCATCTACTCACCA
L61	19-71784	GGACAAGGTCACGCTAGTAAAGGT	TAGGTGAGATTATCGTTCGAGCAG
L62	19-461680	CTAGCCACACGCTCTATCCACAG	GTCTGCTGAGGCTCGAAATGAT
L65	20-447203	TGTCGAGCTTGTTATATTAACGGAA	AACACCATTACCTACAGTCTCGTCG

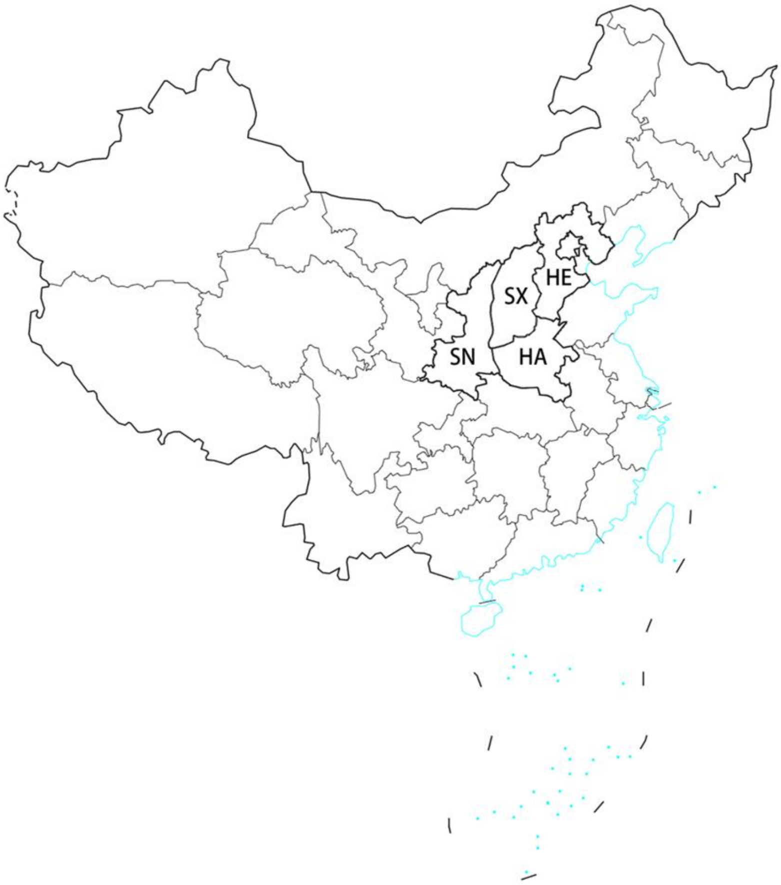

Figure S1. Map illustrating the location of four provinces in which *Setosphaeria turcica* isolates were collected. The blue regions denote coastlines or islands. HA = Henan province, HE = Hebei province, SX = Shanxi province, SN = Shaanxi province.

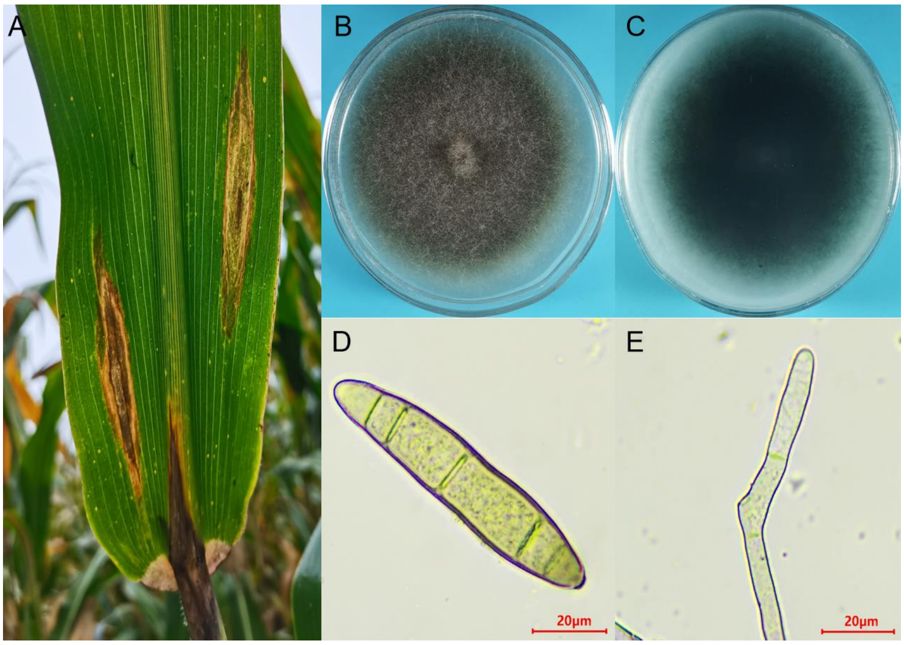

Figure S2. Northern corn leaf blight and *Setosphaeria turcica*. (**A**) Typical symptoms of northern corn leaf blight. (**B**) The fungal colony (upper surface). (**C**) The fungal colony (lower surface). (**D**) Conidium. (**E**) Conidiophore.
